# Team Identification as a Psychological Resource: A Differentiated Model of Nurse Resilience and Team Effectiveness in an Underground Hospital Facility

**DOI:** 10.3390/healthcare14213746

**Published:** 2026-11-03

**Authors:** Nasra Idilbi, Sarit Rashkovits, Jalal Tarabeia, Nasra Abdelhadi, Tammy Porat-Packer, Gizell Green

**Affiliations:** 1 Department of Nursing, The Max Stern Yezreel Valley College, Emek Yezreel 1930600, Israel; 2 Nursing Research Unit, Galilee Medical Center, Nahariya 2210001, Israel; 3 Department of Health Systems Management, The Max Stern Yezreel Valley College, Emek Yezreel 1930600, Israel; 4 Nursing Management, Galilee Medical Center, Nahariya 2210001, Israel; 5 Faculty of Medicine and Health Sciences, Tel Aviv University, Tel Aviv 6902068, Israel; 6 Shoham Medical Center, Pardes Hanna-Karkur 3707382, Israel

**Keywords:** team identification, nurse resilience, team effectiveness, social resources, healthcare workforce, extreme environments, underground hospital

## Abstract

**Highlights:**

**What are the main findings?**

• Team identification was indirectly associated with resilience through affective identification, hope, and optimism.
• Affective identification was linked to personal resources, whereas behavioral identification was linked to team effectiveness.

**What are the implications of the main findings?**

• Different team-identification dimensions may support different aspects of resilience and team functioning.
• Strengthening team belonging and coordinated behaviors may support healthcare staff in high-demand settings.

**Abstract:**

Background: Healthcare professionals working under extreme and constrained conditions face sustained demands that may challenge psychological well-being and team functioning. Team identification may serve as an important social resource for maintaining resilience and coordinated functioning. Objectives: This study examined whether cognitive, affective, and behavioral dimensions of team identification were differentially associated with nurse resilience and perceived team effectiveness during deployment in an underground hospital facility during a national emergency. Methods: A concurrent mixed-methods, cross-sectional study was conducted among nurses and nursing assistants deployed to an underground hospital facility (N = 269). Participants completed measures of team identification, hope, optimism, resilience, and perceived team effectiveness. Structural equation modeling tested a division-of-labor model in which cognitive identification was associated with affective and behavioral identification; affective identification was associated with hope and optimism, which were associated with resilience; and behavioral identification was associated with perceived team effectiveness. A fully crossed model examined the unique contributions of affective and behavioral identification. Open-ended responses were analyzed thematically. Results: Cognitive team identification was indirectly associated with resilience through affective identification and the personal resources of hope and optimism, but was not directly associated with resilience or perceived team effectiveness. In the fully crossed model, affective identification was uniquely associated with hope and optimism, whereas behavioral identification was uniquely associated with perceived team effectiveness. Qualitative findings highlighted emotional support, shared belonging, and coordinated action as important features of functioning in the constrained environment. Conclusions: Team identification may function as a multidimensional social resource for healthcare professionals working under extreme conditions, with distinct affective and behavioral pathways contributing to resilience and perceived team functioning. These findings highlight the potential importance of team-based social resources for workforce well-being and effective functioning during disruptive or constrained healthcare operations. Implications for Practice: Healthcare managers and nursing leaders should consider strategies that strengthen team belonging, emotional connection, and coordinated team behaviors to support workforce resilience and team functioning in high-demand and emergency settings. Longitudinal and intervention studies are warranted.

## 1. Introduction

Providing healthcare in extreme organizational and environmental contexts—such as pandemics, natural disasters, and geopolitical conflicts—poses major operational challenges for healthcare systems globally [1]. Such crises may require the rapid deployment or relocation of clinical services to unconventional environments, where healthcare professionals must adapt to constrained resources, unfamiliar workflows, altered physical structures, and prolonged uncertainty. In some settings, this may include relocating healthcare services to reinforced or underground facilities, creating additional environmental and organizational demands.

Within these high-stakes environments, nurses often serve as frontline providers responsible for maintaining continuity of care and patient safety under prolonged adversity. Understanding nurses’ psychological experiences and capacity to maintain professional functioning is important not only for individual well-being but also for healthcare system resilience [1]. Recent evidence indicates that resilience and social support are important protective resources for nurses’ psychological well-being, highlighting the need to examine both individual and social resources during acute and prolonged crises [2].

Nursing research has documented individual coping strategies and personal resilience among frontline nurses during crises [1]. However, resilience is not solely an individual phenomenon; healthcare professionals work within interdependent teams in which social relationships, shared identities, and mutual support may provide additional resources when individual resources are depleted. Shared social identity can facilitate mutual support, cooperation, and collective resilience [3]. During the COVID-19 pandemic, stronger identification with one’s team was associated with lower stress and burnout, particularly among nurses and other lower-status healthcare workers [4]. Social and team identification has also been linked to clinically relevant processes, including trust, communication, leadership, and coordinated team functioning [5,6].

Despite this emerging evidence, an important gap remains. Existing studies have largely examined team or social identification as a global construct or focused on outcomes such as stress and burnout. Less is known about whether different dimensions of team identification serve distinct psychological and team-functioning roles during extreme healthcare conditions. This distinction may be particularly important in prolonged organizational crises, where a sense of belonging may influence both personal coping and perceptions of collective functioning.

We therefore conceptualize team identification as a potential social resource for nurses and nursing assistants working under highly constrained conditions. Group identification can be differentiated into cognitive, affective, and behavioral dimensions, reflecting social categorization, interpersonal attraction, and behavioral interdependence [7]. Consistent with the Social Cure framework, identification with social groups may provide social resources that support health and well-being [8,9]. Similarly, Conservation of Resources (COR) theory [10] proposes that resources can accumulate and interact within resource caravans [11]. Thus, team identification may function as a social resource linked to additional personal resources within the nurse’s resource caravan.

We examine two complementary psychological and team-functioning outcomes. Personal resilience reflects the capacity to maintain or regain psychological functioning under stress or adversity [12], whereas perceived team effectiveness reflects confidence in the team’s ability to coordinate and perform effectively under demanding conditions. Together, these outcomes capture complementary aspects of individual and collective functioning within the same crisis environment.

To examine how team identification may relate to these outcomes, we conceptualize it as a differentiated rather than unitary construct [7]. The tripartite model distinguishes cognitive identification as recognizing oneself as a group member, affective identification as emotional attachment and belonging, and behavioral identification as interdependence and coordinated engagement toward group goals. Integrating this model with Self-Categorization Theory [13], we propose that cognitive identification may provide a foundation for affective connection and coordinated team behavior, particularly under conditions of shared demands and interdependence.

The first pathway involves affective identification, reflecting emotional bonding and belonging within the team [7]. Such connection may support positive psychological resources during adversity. Hope [14] and optimism [15] represent future-oriented resources that may contribute to resilience, consistent with broaden-and-build theory [16], which proposes that positive psychological states can build enduring personal resources. Thus, affective team identification may be associated with hope and optimism, which may in turn contribute to personal resilience.

The second pathway involves behavioral identification, reflecting interdependence, coordinated engagement, mutual reliance, and contribution to shared goals [7]. In crisis conditions, healthcare delivery depends on coordinated action among interdependent professionals. Behavioral identification may therefore be particularly relevant to nurses’ perceptions of their team’s ability to function effectively under pressure, consistent with evidence linking team identification to coordination and collective functioning [6].

This differentiated perspective is particularly relevant in wartime healthcare settings. Research among nurses exposed to war has identified resilience and perceived social support as protective factors, underscoring the importance of individual and interpersonal resources during prolonged conflict [17]. However, the specific contribution of team identification, and whether its affective and behavioral dimensions serve different functions, remains insufficiently examined.

Using structural equation modeling, this study examines differentiated pathways of team identification among nurses and nursing assistants working in fortified underground hospital facilities during a prolonged national crisis. Integrating the Social Cure perspective, COR theory, and the tripartite model of group identification, we examine how distinct dimensions of team identification relate to personal resilience and perceived team effectiveness, and whether hope and optimism serve as personal-resource pathways linking affective identification to resilience.

Based on this theoretical integration, the study tests the following direct and indirect pathways.

### 1.1. Direct Relationships

**H1:**  *Cognitive team identification will be positively associated with affective team identification.*

**H2:**  *Cognitive team identification will be positively associated with behavioral team identification.*

**H3:**  *Affective team identification will be positively associated with nurses’ hope.*

**H4:**  *Affective team identification will be positively associated with nurses’ optimism.*

**H5:**  *Nurses’ hope will be positively associated with nurse resilience.*

**H6:**  *Nurses’ optimism will be positively associated with nurse resilience.*

**H7:**  *Behavioral team identification will be positively associated with perceived team effectiveness.*

### 1.2. Mediation Hypotheses

**H8:**  *Cognitive team identification will be indirectly associated with nurse resilience through affective team identification and hope.*

**H9:**  *Cognitive team identification will be indirectly associated with nurse resilience through affective team identification and optimism.*

**H10:**  *Cognitive team identification will be indirectly associated with perceived team effectiveness through behavioral team identification.*

## 2. Materials and Methods

### 2.1. Study Design

This study employed a concurrent mixed-methods design [18], in which quantitative and qualitative data were collected concurrently using a single structured questionnaire that incorporated both closed-ended and open-ended items. The quantitative component was used to examine the hypothesized associations and structural paths among the study variables, whereas the qualitative component was used to provide contextual insight into participants’ experiences of delivering frontline clinical care within the constrained subterranean environment. The integration of the two strands was intended to provide a more comprehensive understanding of the relationships examined in the quantitative model and the contextual factors underlying participants’ experiences.

### 2.2. Setting and Participants

The study was conducted among frontline nurses and nursing assistants providing direct patient care within a large acute-care medical center in Israel. At the end of 2023, due to an emergency situation, multiple inpatient departments were relocated to a fortified underground complex. Importantly, this subterranean facility had not been originally designed for prolonged inpatient hospitalization, thereby creating a highly constrained clinical and environmental setting.

The underground setting was characterized by multiple environmental and operational challenges, including predominantly artificial lighting, high humidity, overcrowding in patient care areas and staff workstations, elevated noise levels, and limited access to natural daylight and fresh air. These physical constraints were accompanied by substantial disruptions to established nursing workflows and clinical care processes. Nursing teams were required to adapt rapidly to unfamiliar ward layouts, modified physical structures, and limited resources. Furthermore, following the discharge of patients who were considered clinically stable or less medically dependent, the remaining patient population was characterized by a relatively high level of medical acuity and dependency. Consequently, nurses were required to manage increased clinical complexity and workload while maintaining routine standards of care and patient safety under emergency conditions.

Eligible participants were nurses and nursing assistants who had been actively working and providing direct patient care in the relocated underground units for at least two consecutive months before data collection. This criterion was established to ensure sufficient exposure to the environmental and clinical conditions of the underground setting. Following formal approval from the institutional ethics committee, paper questionnaires were distributed to eligible participants by trained research assistants and returned anonymously to a designated secure collection box. Participants were excluded if they were healthcare professionals other than nurses or nursing assistants or if they had worked in the underground facility for less than two months.

The Institutional Ethics Committee approved the study on 19 March 2024 (Approval No. NHR-044-24). Quantitative and qualitative data were collected from early April through the end of July 2024. A total of 300 eligible nurses and nursing assistants completed the questionnaire. Following data screening, 269 participants with complete data for all variables included in the structural path analyses constituted the final analytic sample.

### 2.3. Research Instruments

The study questionnaire included closed-ended and open-ended questions. Each quantitative construct was reported by the individual nurse or nursing assistant and analyzed at the individual level. For the established measurement instruments, the original item wording, response scales, and scoring procedures were retained without modification. To focus responses on experiences specific to the underground deployment rather than on stable general dispositions, a contextual instruction was provided at the beginning of each instrument, directing participants to answer the items with reference to their work and experiences during the underground deployment. The complete questionnaire, including these contextual instructions, was reviewed by experts for clarity, relevance, and appropriateness to the study population and setting and was pilot-tested with healthcare personnel before the main data collection. Feedback from the expert review and pilot testing was used to refine the questionnaire instructions and presentation where necessary.

(1) Hope: Adapted from the Dispositional Hope Scale [19], this measure consists of eight items assessing hope across two subscales (four Pathways and four Agency items). Items were rated on a 4-point scale (1 = definitely false, 2 = mostly false, 3 = mostly true, 4 = definitely true). An example contextualized item is: “During my time working in the underground facility, I could think of many ways to get out of the crisis.” Cronbach’s alpha in the current study was 0.86.
(2) Optimism: Adapted from the Life Orientation Test (Lot) [15,20], this measure consists of eight items assessing situational optimism, rated on a 5-point Likert-type scale ranging from 1 (strongly disagree) to 5 (strongly agree). An example contextualized item is: “During my time working in the underground facility, I felt I was always optimistic about my future.” One item (“I rarely count on good things happening to me”, reverse-scored) was excluded from the operational score because it showed the weakest corrected item-total correlation (r = 0.13, vs. 0.19–0.52 for the other items) and was essentially uncorrelated with the positively worded items; this decision was based on the item’s performance in the present sample rather than made a priori. Cronbach’s alpha for the resulting 7-item score was 0.70 (vs. 0.65 for the full 8-item version).
(3) Team Identification: Based on the tripartite Group Identification Scale [7], this instrument consists of 11 items representing cognitive, affective, and behavioral identification. An example cognitive-identification item is: ‘During my time working in the underground facility, I thought of this team as part of who I am.’ An example affective-identification item is: ‘During my time working in the underground facility, I felt a strong sense of emotional bonding with my team members.’ An example behavioral-identification item is: ‘During my time working in the underground facility, I felt that this team coordinated effectively to achieve collective goals.’ Items are rated on a 7-point Likert scale ranging from 1 (strongly disagree) to 7 (strongly agree); some items were reverse-scored. Cronbach’s alpha was 0.88 for the overall scale and 0.68, 0.75, and 0.75 for the cognitive, behavioral, and affective facets, respectively. The comparatively low reliability of the cognitive facet is considered further in Section 4.2.
(4) Personal Resilience: Assessed using the Resilience Scale-14 [21], comprising 14 items rated on a 7-point Likert scale ranging from 1 (strongly disagree) to 7 (strongly agree). An example contextualized item is: “During my time working in the underground facility, I felt that I usually manage to cope.” Cronbach’s alpha in the current study was 0.94.
(5) Perceived Team Effectiveness: Assessed using a four-item questionnaire developed by the research team based on the literature of Tjosvold [22]. The items assessed team output, efficiency, work morale, and patient safety and were contextualized to the underground facility. Responses were rated on a 5-point Likert scale, with higher scores indicating greater perceived team effectiveness. Cronbach’s alpha was 0.80.
(6) Sociodemographic and Professional Characteristics: included age, sex, marital status, religion and religiosity, education, professional role, department, employment percentage, and tenure in the profession and department.
(7) Qualitative Open-Ended Items: Three open-ended questions were included to explore participants’ experiences and identify factors that promoted or hindered coping while working in an underground facility: (a) Which factors have helped you cope with working in the underground facility? (b) Which factors do you believe have hindered your ability to cope with working in the underground facility? (c) Is there anything else you would like to share about your experience working in the underground facility?

Of the 269 participants, 242 (90.0%) provided responses to the open-ended questions, which were included in the thematic analysis.

### 2.4. Statistical Analysis

Descriptive statistics and Pearson correlations were calculated for all study variables. Path models were estimated using maximum likelihood on a common analytic sample within a Structural Equation Modeling (SEM) framework. Cases with missing data on any study variable were excluded listwise, yielding a common analytic sample of N = 269 (*n* = 31 excluded, 10.3% of the eligible sample of 300). Among the study variables, education showed the highest proportion of missing values (*n* = 14, 4.7%), followed by hope and perceived team effectiveness (*n* = 8, 2.7%); all other study variables had less than 2% missingness. All structural equation models were estimated in Python (version 3.12.3) using the semopy package (version 2.3.11) [23]. Specific indirect associations corresponding to Hypotheses 8–10 were tested using 2000 bootstrap resamples and 95% percentile confidence intervals. Additional models were estimated that added direct paths from cognitive identification to the distal outcomes, to test whether mediation was full or partial.

The hypothesized division-of-labor model (affective identification associated with hope and optimism; behavioral identification associated with perceived team effectiveness) was first compared with a more parsimonious model in which overall perceived identification was entered as a single composite. The hypothesized division-of-labor structure was also compared with a fully crossed alternative, in which the affective and the behavioral identification facets each predicted all three downstream variables, using a nested chi-square difference test together with AIC and BIC; this comparison was designed to test whether these two identification facets show the expected differentiated, rather than uniform, pattern of association with the study’s two outcome domains.

Education was entered as a covariate of resilience on prior literature linking educational attainment to resilience [24], and parallel mediators were allowed correlated residuals. Model fit was evaluated using χ^2^, CFI, TLI, and RMSEA. Common method variance (CMV) was evaluated using Harman’s single-factor test and the marker-variable technique [25].

### 2.5. Qualitative Analysis

Qualitative data from the open-ended questions were analyzed using inductive content analysis [26] to identify recurring patterns and themes. Two researchers independently reviewed and coded responses, developed categories, and organized them into overarching themes. Interpretations were compared throughout the process, and discrepancies were resolved through discussion and consensus; a third researcher reviewed the coding and contributed to the final refinement of themes [27]. The qualitative findings were integrated as contextual evidence concerning nurses’ experience of identification, personal resources, resilience, and perceived team effectiveness in the underground facility.

The quantitative and qualitative data were analyzed independently and concurrently. Integration occurred at the interpretation stage, when the qualitative themes were compared with the quantitative findings to contextualize and elaborate on the observed patterns.

### 2.6. Ethical Considerations

The study protocol was reviewed and approved by the Institutional Ethics Committee of the Galilee Medical Center (Approval No. NHR-044-24) on 19 March 2024, and was conducted in accordance with the Declaration of Helsinki and the approved ethical procedures.

Following approval, a designated member of the research team distributed the questionnaires to eligible healthcare professionals. Before participation, participants were informed about the study purpose and procedures, the voluntary and anonymous nature of participation, confidentiality, and their right to decline without consequences.

In accordance with the ethics committee-approved procedure, the requirement for a separately signed written informed consent form was waived. Instead, informed consent was obtained through voluntary completion and return of the anonymous questionnaire, which indicated consent to participate. Participants were not asked to provide identifying information or a reason for non-participation. Completed questionnaires were placed in sealed envelopes and deposited in a secure collection box, and the data were accessible only to authorized members of the research team.

## 3. Results

### 3.1. Participants’ Socio-Demographic and Professional Characteristics

Participants’ mean age was 42.5 ± 10.6 years (range: 22–66). The mean full-time equivalent percentage was 93.7 ± 10.0 (range: 20–100). Participants had a mean of 16.4 ± 11.2 years of professional experience in nursing (range: 0–44), and a mean of 9.6 ± 8.5 years of experience within the same department (range: 0–40) (Table 1).

Most participants were female (202, 77.4%) and married (214, 79.9%). The most common religious affiliations were Jewish (128, 48.9%), Muslim (60, 22.9%), and Christian (59, 22.5%). Over half of the participants (145, 53.9%) held a bachelor’s degree, and 75 (27.9%) attained a master’s degree or higher. The participants comprised 245 nurses (92.1%) and 21 nursing assistants (7.9%). The most common departments were surgical (87, 34.0%) and internal medicine (81, 31.6%) (Table 1).

### 3.2. Descriptive Statistics, Reliabilities, and Correlations Among Study Variables

Table 2 presents mean, standard deviations, internal consistency reliabilities (Cronbach’s α, on the diagonal), and zero-order correlations among the study variables. Reverse-worded items were recoded so that higher scores consistently reflected higher standing on each construct. Perceived Team Effectiveness was not significantly correlated with Nurse Resilience (r = 0.12, n.s.), supporting their treatment as distinct domains within nurses’ experience. Notably, cognitive identification showed only weak zero-order correlations with optimism (r = 0.12, *p* < 0.05) and nurse resilience (r = 0.13, *p* < 0.05), the weakest among the identification facets, consistent with its hypothesized role as an upstream facet whose associations with these distal outcomes are expected to be carried by the affective and behavioral facets.

### 3.3. Structural Path Coefficients and Hypothesis Testing

The hypothesized model (division-of-labor structure) showed a near-perfect fit to the data (χ^2^(20) = 16.69, *p* = 0.673; CFI = 1.00; RMSEA ≈ 0). A CFI marginally exceeding 1.00 can occur when a model’s chi-square value falls below its degrees of freedom, and reflects a very close correspondence between the hypothesized model and the observed covariance structure rather than a modeling problem.

**Figure 1:**
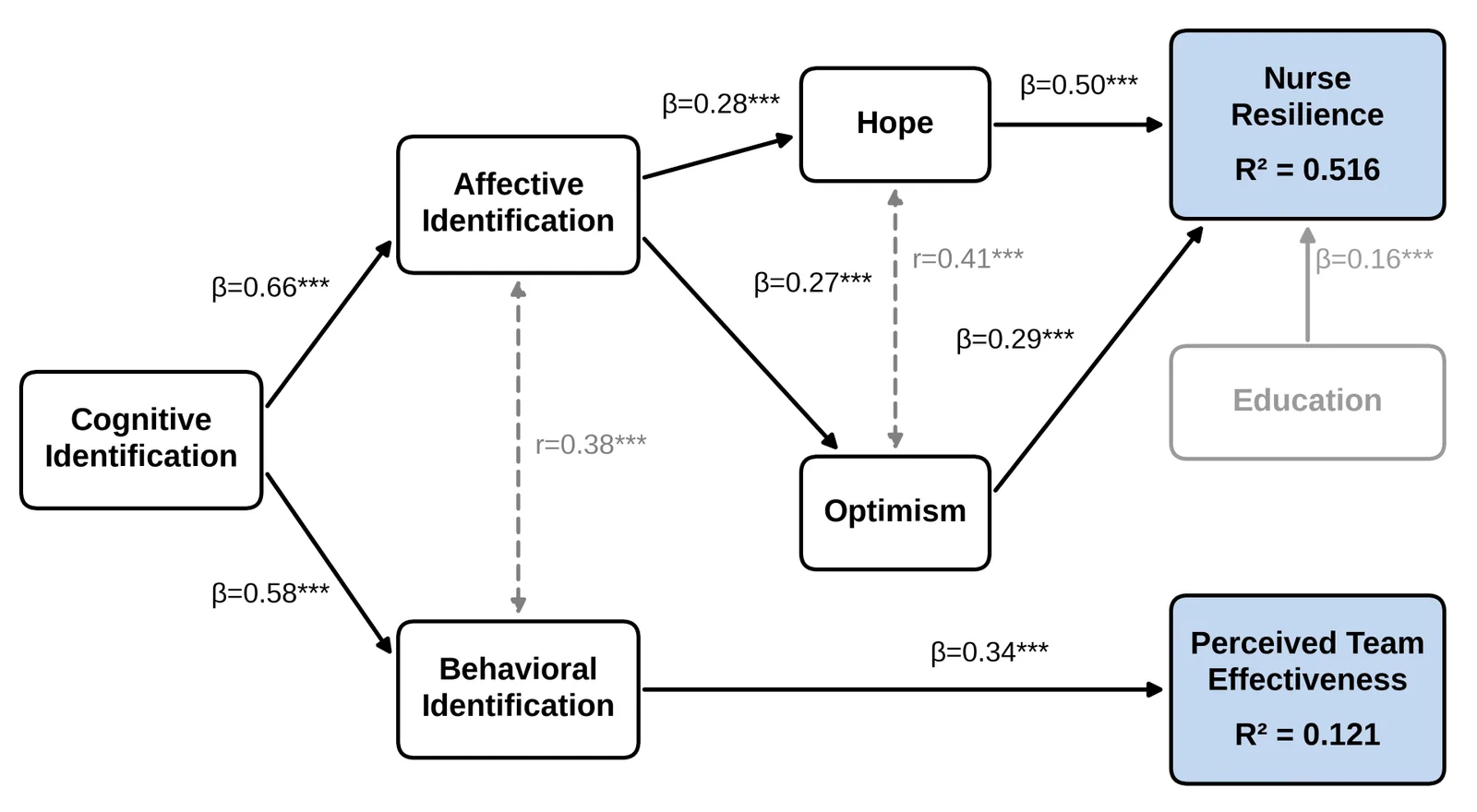
Estimated path model with standardized coefficients (Hypothesized model—division of labor). Note. Standardized coefficients are shown. *** *p* < 0.001. Dashed double-headed arrows represent correlated residuals. Blue boxes denote outcome variables (R^2^ shown). Education is entered as a covariate of Nurse Resilience.

Table 3 presents the path coefficients for the main hypothesized division-of-labor model (see also Figure 1).

Consistent with H1 and H2, cognitive identification was positively associated with both affective identification (β = 0.655, *p* < 0.001) and behavioral identification (β = 0.579, *p* < 0.001). Supporting H3 and H4, affective identification was positively associated with both hope (β = 0.281, *p* < 0.001) and optimism (β = 0.272, *p* < 0.001). In turn, and consistent with H5 and H6, both hope (β = 0.499, *p* < 0.001) and optimism (β = 0.294, *p* < 0.001) were positively associated with nurse resilience. Education was also significantly associated with resilience (β = 0.155, *p* < 0.001). Supporting H7, behavioral identification was positively associated with perceived team effectiveness (β = 0.343, *p* < 0.001).

As can be seen in Table 3 (indirect effects section), the hypothesized indirect paths were also supported. Bootstrap analyses (2000 resamples) supported H8: affective identification and hope serially carried the indirect association between cognitive identification and nurse resilience (indirect effect = 0.092, 95% CI [0.052, 0.137]). H9 was also supported: affective identification and optimism serially carried the indirect association between cognitive identification and nurse resilience (indirect effect = 0.053, 95% CI [0.027, 0.082]). Finally, supporting H10, behavioral identification carried the indirect association between cognitive identification and perceived team effectiveness (indirect effect = 0.199, 95% CI [0.123, 0.276])—the largest standardized indirect effect anywhere in the study. Taken together, all ten hypotheses were supported (Table 3).

To test whether mediation was full or partial, direct paths from cognitive identification to the distal outcomes (not depicted in Figure 1) were added to the model. The direct path from cognitive identification to nurse resilience was non-significant (β = 0.004, *p* = 0.931), as was the direct path to perceived team effectiveness (β = 0.05, *p* = 0.452). These results are consistent with an indirect-only pattern: cognitive identification showed no independent association with either distal outcome beyond its indirect routes through affective and behavioral identification, consistent with its hypothesized role as the upstream facet associated with the other two.

### 3.4. Model Fit: The Hypothesized Division-of-Labor Versus Overall Identification Models

To verify the utility of differentiating among the cognitive, affective, and behavioral identification facets—as proposed in our hypothesized division-of-labor model—we compared this model with an alternative in which overall identification was entered as a single composite variable.

**Figure 2:**
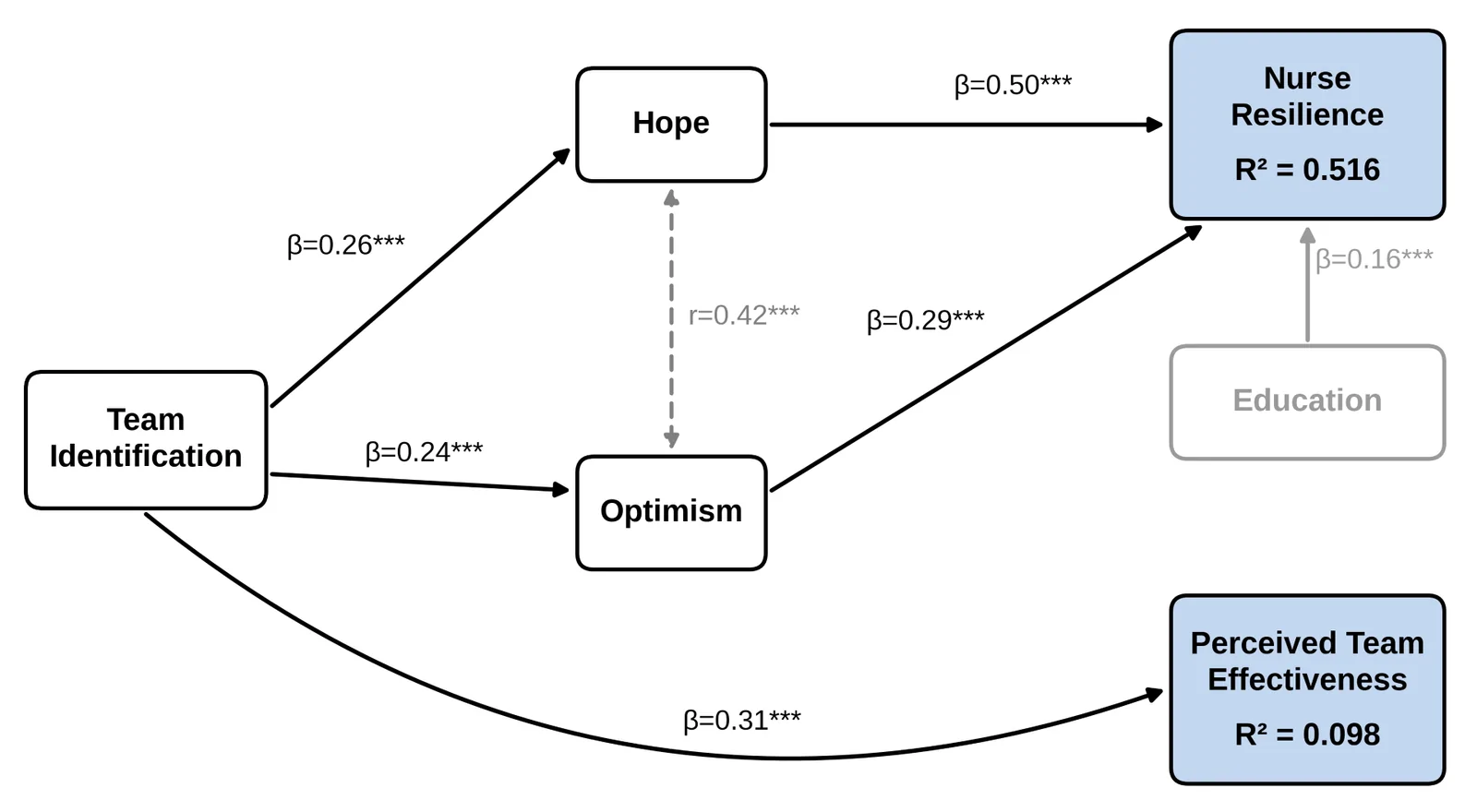
Estimated path model (Alternative parsimonious model—Overall Identification) with standardized coefficients. Note. Standardized coefficients are shown. *** *p* < 0.001. The dashed double-headed arrow represents the correlated residual between Hope and Optimism. Blue boxes denote outcome variables (R^2^ shown). Education is entered as a covariate of Nurse Resilience.

Table 4 presents the path coefficients for this parsimonious alternative model (overall identification; see also Figure 2), including its indirect path coefficients.

As shown in Table 5, both models fit the data well (overall identification Model: χ^2^(9) = 9.49, *p* = 0.394, CFI = 0.998, RMSEA = 0.014; Model 2: χ^2^(20) = 16.69, *p* = 0.673, CFI = 1.00, RMSEA ≈ 0). Because the overall identification Model requires fewer estimated parameters, it also showed lower AIC and BIC values; however, as shown in Table 6, the division-of-labor structure explained more variance in perceived team effectiveness, supporting the added value of differentiating among the three identification facets. For nurse resilience, overall identification Model explained essentially the same proportion of variance as Model 2 (51.6% vs. 51.6%)—an equivalence that follows directly from both models specifying an identical predictive equation for resilience (hope, optimism, and education), such that any difference between the models in how these predictors are themselves generated upstream does not alter the variance explained in resilience itself. For perceived team effectiveness, however, the overall identification Model explained a noticeably smaller proportion of variance than Model 2 (9.8% vs. 12.1%—roughly one-fifth less variance explained in relative terms).

### 3.5. Division-of-Labor Versus Fully Crossed Model Comparison

To further confirm our hypothesized model—in which affective identification leads to the personal resources of optimism and hope, while behavioral identification leads to perceived team effectiveness—this division-of-labor structure was tested against a fully crossed alternative in which both affective and behavioral identification predicted all three second-stage variables.

**Figure 3:**
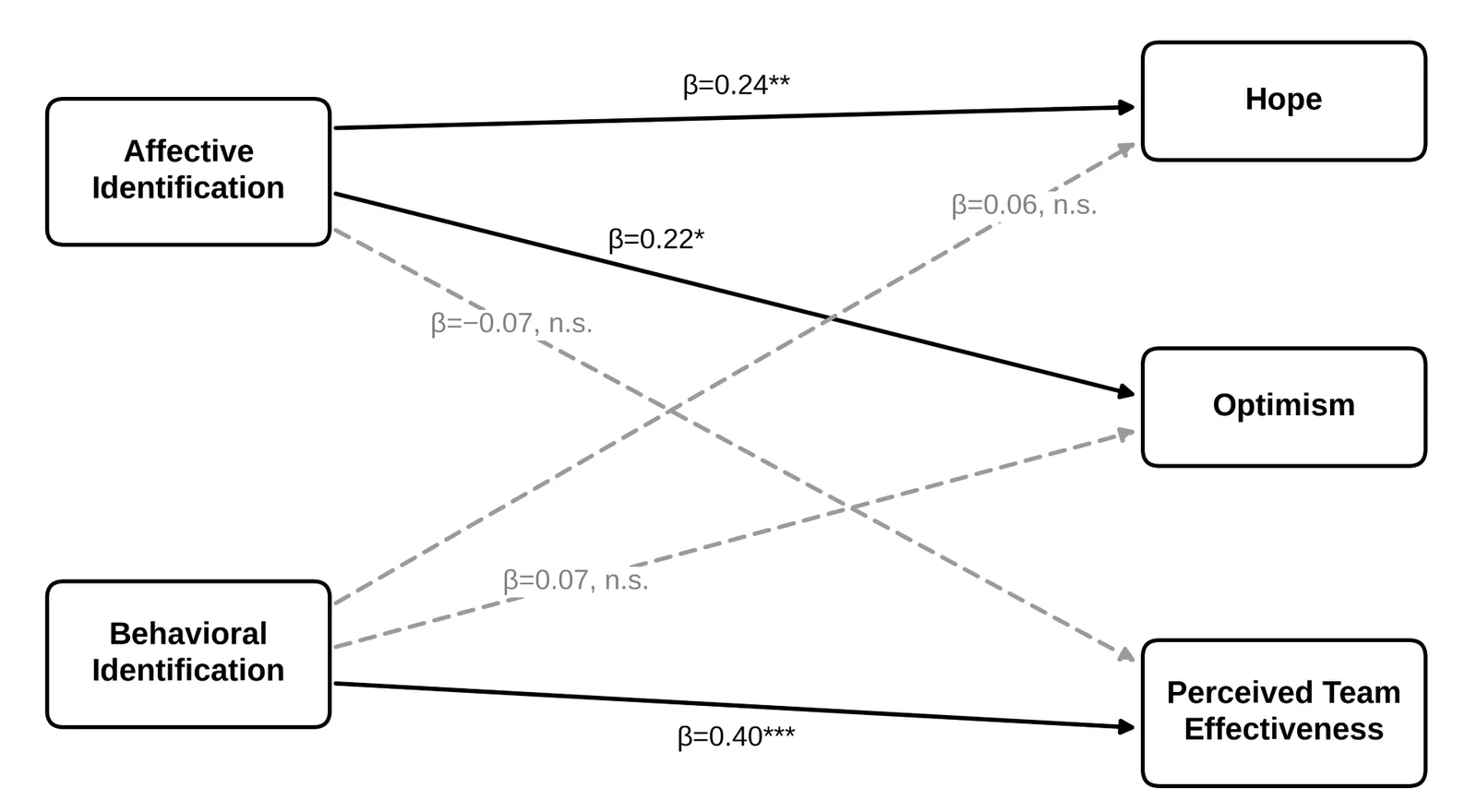
Fully crossed alternative model with standardized path coefficients. Note. Solid black arrows indicate significant paths; dashed gray arrows indicate non-significant paths (n.s.). ** *p* < 0.01, *** *p* < 0.001.

The results, as seen in Figure 3, show that in the fully crossed model, affective identification was the only significant statistical predictor of the personal resources—hope (β = 0.239, *p* = 0.008) and optimism (β = 0.218, *p* = 0.015), while behavioral identification’s unique contribution to each was non-significant (β = 0.056, *p* = 0.533; β = 0.072, *p* = 0.422, respectively). Conversely, behavioral identification was the only significant statistical predictor of perceived team effectiveness (β = 0.395, *p* < 0.001), while affective identification’s unique contribution was non-significant and trivial in size (β = −0.069, *p* = 0.431).

A chi-square difference test confirmed that constraining these three non-significant paths to zero (i.e., adopting the division-of-labor structure) did not significantly worsen fit relative to the fully crossed model, Δχ^2^(3) = 1.35, *p* = 0.717, and the more parsimonious division-of-labor model was favored by both AIC (Δ = −6.01) and BIC (Δ = −16.79).

### 3.6. Common Method Variance Checks

Harman’s single-factor test, conducted on the 45 items comprising the five study scales, showed that the first unrotated factor accounted for 28.8% of the total variance, below the conventional 50% threshold for a single dominant factor. The marker-variable technique [26], using participant religiosity as the marker (smallest observed correlation with a substantive variable: r = −0.001, with Perceived Team Effectiveness), left all key correlations virtually unchanged after adjustment. Together with the selective pattern of associations, these checks suggest that common method variance is unlikely to fully account for the findings, although it cannot be ruled out by statistical tests alone.

Cognitive identification showed no direct association with either distal outcome (Section 3.3), consistent with a pattern in which its associations with resilience and perceived team effectiveness ran only through two functionally distinct pathways—an affective pathway linked to personal resilience via hope and optimism, and a behavioral pathway linked independently to perceived team effectiveness (Section 3.5). This same specificity is consistent with why collapsing identification into a single composite corresponded to reduced explanatory power for perceived team effectiveness, while resilience remained unaffected, since both models rely on an identical downstream predictive equation regardless of which upstream facet is associated with hope and optimism (Section 3.4). This selective, non-uniform pattern of associations is itself informative with respect to common method concerns: a shared-method artifact would be expected to inflate correlations broadly across variables, whereas the sharply differentiated pattern observed here—significant, specific associations alongside clearly non-significant ones—is more consistent with substantive, facet-specific relationships than with a generalized reporting bias (Section 3.6).

### 3.7. Qualitative Results

Content analysis yielded three themes that complemented the quantitative division-of-labor model.

**1.**  **Team integration as a foundation for cognitive and affective identification.**

The integration of previously separate departments created opportunities for shared experiences, emotional disclosure, and mutual support. As one nurse explained: “The new move made us meet a new team that I didn’t know before; it allowed us to share experiences and vent our feelings.” This theme illustrates how working under a shared reality may strengthen employees’ sense of belonging to the team and their emotional identification with it.

**2.**  **Affective identification as a source of hope and optimism.**

Emotional support and mutual listening were described as strengthening positive psychological resources. One participant noted: “They listened to me, and I could share my feelings with them… I felt more optimistic after the talks.” This theme adds experiential depth to the indirect pathways from affective identification through hope and optimism to resilience.

**3.**  **Behavioral identification as a resource for team effectiveness.**

Participants emphasized practical assistance, cooperation, and mutual reliability. As one nurse stated: “The team is cohesive… we have each other’s back.” This theme reinforces the quantitative association between behavioral identification and perceived team effectiveness and illustrates how identification with the team may be expressed through concrete supportive actions during a crisis.

## 4. Discussion

This study examined how team identification—differentiated into cognitive, affective, and behavioral components [7]—was associated with nurses’ and nursing assistants’ personal resilience and perceived team effectiveness in an underground hospital facility during a prolonged crisis. The findings provide support for a differentiated “division-of-labor” pattern in which distinct components of team identification were associated with different psychological and team-related outcomes. Cognitive identification was associated with both affective and behavioral identification, while affective identification was associated with hope and optimism, which were in turn associated with personal resilience. In contrast, behavioral identification was associated with perceived team effectiveness. A direct model comparison further indicated that differentiating team identification into its constituent components provided a better representation of perceived team effectiveness than treating identification as a single overall construct. Taken together, these findings suggest that team identification may function as a multidimensional psychological and interpersonal resource in highly constrained healthcare environments, with different components potentially serving different functions.

The association of cognitive identification with both affective and behavioral identification is consistent with Self-Categorization Theory [7,13] tripartite model. From this perspective, recognizing oneself as a member of a particular team may provide a basis for developing stronger emotional attachment to the team and greater involvement in coordinated team activity. In the present setting, nurses and nursing assistants were working within a shared and unfamiliar environment characterized by substantial physical and operational constraints. Such conditions may have increased the salience of team membership, as staff depended on colleagues to coordinate care, adapt to changing circumstances, and maintain continuity of clinical work. This interpretation is also broadly consistent with research suggesting that shared social identity can become particularly salient under conditions of collective adversity [3]. However, because the present study was cross-sectional, the observed associations should not be interpreted as evidence that cognitive identification causes affective or behavioral identification.

The affective pathway provides further support for the potential psychological relevance of team identification in crisis healthcare environments. Affective identification was associated with hope and optimism, which were subsequently associated with personal resilience. This pattern is consistent with the Social Cure perspective, which proposes that meaningful group memberships can provide psychological resources that support health and well-being [8,9]. It is also compatible with the broaden-and-build theory [16], according to which positive psychological states may broaden individuals’ cognitive and behavioral repertoires and contribute to the development and maintenance of adaptive resources. In a prolonged emergency setting, emotional attachment to the team may therefore provide a social context within which positive expectations and future-oriented beliefs can be maintained despite ongoing occupational demands.

This interpretation is broadly consistent with previous research among healthcare professionals. Stronger work-team identification has been associated with lower stress and burnout among frontline healthcare workers during an acute health crisis [4], while previous studies have linked hope and optimism with resilience among nurses and other healthcare professionals exposed to occupational stress [28,29]. The present findings extend this literature by examining these relationships in a prolonged emergency relocation involving an underground hospital environment and, importantly, by identifying affective identification as the component most closely associated with the positive psychological resources examined. This distinction may be relevant for healthcare organizations seeking to support staff resilience during prolonged periods of operational disruption.

The behavioral pathway showed a different pattern. Behavioral identification was associated with perceived team effectiveness, independent of the affective pathway. This finding is consistent with the conceptualization of behavioral identification as reflecting active involvement and interdependence in group activities [7]. In a highly constrained clinical environment, where patient care depends on rapid coordination, role flexibility, and mutual assistance, the extent to which staff perceive themselves as actively participating in coordinated team processes may be particularly relevant to their evaluation of team effectiveness. This interpretation is broadly compatible with evidence that team processes, including team learning activities, are associated with perceived team effectiveness among nursing teams [30].

Importantly, the fully crossed model provided additional evidence for the differentiated nature of these associations. Affective identification was not significantly associated with perceived team effectiveness after behavioral identification was considered, whereas behavioral identification was not significantly associated with hope or optimism after affective identification was considered. Thus, the findings do not suggest that stronger identification is uniformly beneficial across all outcomes. Rather, they suggest that the specific way in which individuals identify with their team may be relevant to the type of outcome under consideration. This distinction is particularly relevant in healthcare settings, where psychological well-being and team functioning are related but conceptually distinct dimensions of effective clinical work.

Education also emerged as an independent correlate of personal resilience beyond the identification-related pathways. This finding is consistent with previous evidence linking higher educational attainment with greater resilience among nurses during acute crises [25], although this relationship has not been consistently observed across nursing populations and occupational contexts. One possible explanation is that educational attainment may be associated with greater access to professional knowledge, problem-solving strategies, perceived competence, or other personal resources that support adaptation under challenging conditions. Nevertheless, these mechanisms were not directly examined in the present study. Because education generally precedes the crisis period examined, reverse causation is less plausible; however, the cross-sectional design does not allow an independent causal effect of education on resilience to be established.

Although the study was conducted in an extreme subterranean setting, the findings may have relevance to conventional clinical environments experiencing severe strain, such as periods of extreme understaffing or future pandemics. In such settings, affective identification may represent a potentially important social resource for supporting hope and optimism, while behavioral identification may contribute to coordinated team functioning.

### 4.1. Implications for Healthcare Practice

The findings have several potential implications for healthcare management and nursing practice in crisis and resource-constrained environments. First, the results suggest that approaches designed to strengthen team identification may benefit from distinguishing between its cognitive, affective, and behavioral components rather than treating identification as a single construct. For example, team-based approaches could aim to strengthen a shared sense of team membership while also creating opportunities for meaningful interpersonal connection and active collaboration.

Second, the different associations observed for affective and behavioral identification suggest that approaches targeting staff psychological resources may differ from those aimed at strengthening perceived team functioning. Activities that foster trust, belonging, and emotional connection may help create conditions that support psychological resources such as hope and optimism, whereas structured opportunities for interdependent work, collaborative decision-making, and team learning may be more closely aligned with perceived team effectiveness.

These implications may be particularly relevant in healthcare environments characterized by prolonged disruption, emergency relocation, limited physical resources, or other conditions in which routine structures are compromised. In such settings, strengthening team-based resources may represent a potentially modifiable strategy for supporting both staff well-being and team functioning. However, because the present study was cross-sectional and observational, these findings should not be interpreted as evidence that such approaches improve resilience or team effectiveness. Longitudinal and intervention-based research is needed to determine whether strengthening specific dimensions of team identification can produce these outcomes.

### 4.2. Limitations

Several limitations should be considered when interpreting the findings. First, the study used a cross-sectional, single-timepoint design, with all variables measured concurrently through the same questionnaire. Therefore, the observed associations are consistent with, but do not establish, the temporal sequence proposed in the hypothesized model. The ordering of cognitive, affective, and behavioral identification cannot be determined, and reciprocal or alternative pathways remain plausible.

Second, the reliance on self-report measures introduces the possibility of common method variance and shared-source bias, as participants provided information on both predictor and outcome variables. The marker-variable and Harman’s single-factor analyses reported in the Methods did not suggest that common method variance fully accounted for the observed relationships; however, such statistical procedures cannot completely exclude this source of bias. The differentiated pattern of associations—particularly the association of affective identification with hope and optimism but not perceived team effectiveness, and the association of behavioral identification with perceived team effectiveness but not hope or optimism—provides some evidence against a simple explanation based solely on generalized response tendencies, but does not eliminate the possibility of shared-method effects.

Third, the study was conducted in a single acute-care medical center and within one highly specific emergency context, involving nurses and nursing assistants working in an underground hospital facility. The unique environmental, organizational, and clinical characteristics of this setting may limit the transferability of the findings to conventional hospital environments, other healthcare systems, or different types of disasters and crises. Replication across multiple institutions and crisis contexts is therefore warranted.

A further limitation concerns the relatively low internal consistency of the cognitive identification dimension (Cronbach’s α = 0.68), which may have attenuated or otherwise affected the observed associations involving this dimension.

Finally, the study design does not permit conclusions regarding whether strengthening team identification would lead to improvements in resilience, hope, optimism, or team effectiveness. Future longitudinal studies should examine whether changes in different components of team identification precede changes in psychological and team outcomes. Intervention studies could further determine whether targeted strategies to strengthen affective and behavioral team identification produce measurable improvements in staff well-being, team functioning, and ultimately the quality and safety of healthcare delivery.

### 4.3. Conclusions

Overall, the findings support a differentiated view of team identification in a highly constrained healthcare environment. Cognitive identification was associated with affective and behavioral identification, whereas affective identification was associated with hope and optimism and behavioral identification was associated with perceived team effectiveness. These findings suggest that team identification may represent an important team-level resource for healthcare professionals working under prolonged crisis conditions, with its different components potentially relating to distinct aspects of personal and team functioning. The findings highlight the value of examining team identification as a multidimensional construct rather than as a unitary phenomenon. Longitudinal and intervention-based research is needed to establish the temporal and causal ordering of these relationships and to determine whether strengthening specific dimensions of team identification can contribute to more resilient healthcare teams.

## Figures and Tables

**Table 1:** Participant Demographics and Professional Characteristics (N = 269).

Variable	Category/M ± SD	*n* (%)/Min–Max
**Continuous variables**		
Age (years)	42.5 ± 10.6	22–66
Full-time equivalent percentage	93.7 ± 10.0	20–100
Years of experience in nursing	16.4 ± 11.2	0–44
Years of experience within the same department	9.6 ± 8.5	0–40
**Categorical variables**		
Gender	Female	202 (77.4%)
	Male	59 (22.6%)
Marital Status	Married	214 (79.9%)
	Single	30 (11.2%)
	Divorced	22 (8.2%)
	Widowed	1 (0.4%)
	Other	1 (0.4%)
Religious affiliation	Jewish	128 (48.9%)
	Christian	59 (22.5%)
	Muslim	60 (22.9%)
	Druze	11 (4.2%)
	Other	4 (1.5%)
Highest education level	Bachelor’s degree	145 (53.9%)
	Master’s degree or higher	75 (27.9%)
	Post-secondary level	49 (18.2%)
Professional role	Nurse	245 (92.1%)
	Nurse assistant	21 (7.9%)
Department	Internal medicine	81 (31.6%)
	Surgical	87 (34.0%)
	Maternity	49 (19.1%)
	Pediatrics	31 (12.1%)
	Chronic care	6 (2.3%)
	Other	2 (0.8%)

Note. SD = Standard Deviation; Min = Minimum value; Max = Maximum value; *n* = number of participants; % = percentage of valid responses; percentages may not sum to 100% due to rounding or missing data.

**Table 2:** Means, standard deviations, reliabilities, and intercorrelations among study variables (N = 269).

Variable	M	SD	1	2	3	4	5	6	7	8	9
1. Team Identification (overall)	61.68	11.32	(0.88)								
2. Cognitive Identification	16.07	3.89	0.83 ***	(0.68)							
3. Affective Identification	23.28	4.38	0.92 ***	0.65 ***	(0.75)						
4. Behavioral Identification	22.32	4.56	0.89 ***	0.58 ***	0.76 ***	(0.75)					
5. Hope	3.19	0.49	0.26 ***	0.17 **	0.28 ***	0.24 ***	(0.86)				
6. Optimism	3.78	0.52	0.24 ***	0.12 *	0.27 ***	0.24 ***	0.49 ***	(0.70)			
7. Nurse Resilience	5.79	0.91	0.23 ***	0.13 *	0.25 ***	0.23 ***	0.65 ***	0.54 ***	(0.94)		
8. Perceived Team Effectiveness	12.15	3.95	0.31 ***	0.23 ***	0.23 ***	0.34 ***	0.16 **	0.20 ***	0.12	(0.80)	
9. Education	2.10	0.67	0.07	0.06	0.06	0.07	0.12	0.05	0.23 ***	0.06	—

Note. N = 269. Values on the diagonal (in parentheses) are Cronbach’s α. * *p* < 0.05, ** *p* < 0.01, *** *p* < 0.001.

**Table 3:** Structural Path Coefficients and Indirect (Mediated) Effects, Division-of-Labor Model (N = 269).

Direct Effects				
**Path**	**b (unstd.)**	**β (std.)**	***p***	
Cognitive Identification → Affective Identification	0.737	0.655	<0.001	
Cognitive Identification → Behavioral Identification	0.679	0.579	<0.001	
Affective Identification → Hope	0.031	0.281	<0.001	
Affective Identification → Optimism	0.032	0.272	<0.001	
Hope → Resilience	0.916	0.499	<0.001	
Optimism → Resilience	0.513	0.294	<0.001	
Education → Resilience	0.207	0.155	<0.001	
Behavioral Identification → Perceived Team Effectiveness	0.297	0.343	<0.001	
Affective ↔ Behavioral Identification (residual corr.)	7.521	0.378	<0.001	
Hope ↔ Optimism (residual corr.)	0.103	0.409	<0.001	
**Indirect (Mediated) Effects (bootstrap 95% CIs)**				
**Indirect path**	**b**	**95% CI (unstd.)**	**β**	**95% CI (std.)**
Cognitive → Affective Identification → Hope → Resilience	0.0213	[0.0110, 0.0339]	0.092	[0.052, 0.137]
Cognitive → Affective Identification → Optimism → Resilience	0.0122	[0.0058, 0.0203]	0.053	[0.027, 0.082]
Cognitive → Behavioral Identification → Perceived Team Effectiveness	0.2022	[0.1253, 0.2862]	0.199	[0.123, 0.276]
Total indirect effect on Resilience (sum of first two rows)	0.0335	[0.0192, 0.0499]	—	—

Note. R^2^ (Hope) = 8.2%; R^2^ (Optimism) = 7.8%; R^2^ (Resilience) = 51.6%; R^2^ (Perceived Team Effectiveness) = 12.1%.

**Table 4:** Structural Path Coefficients and Indirect (Mediated) Effects, Overall Identification Model (N = 269).

Direct Effects				
**Path**	**b (unstd.)**	**β (std.)**	***p***	
Identification → Hope	0.011	0.263	<0.001	
Identification → Optimism	0.011	0.242	<0.001	
Hope → Resilience	0.916	0.498	<0.001	
Optimism → Resilience	0.513	0.294	<0.001	
Education → Resilience	0.207	0.155	<0.001	
Identification → Perceived Team Effectiveness	0.107	0.307	<0.001	
Hope ↔ Optimism (residual corr.)	0.106	0.422	<0.001	
**Indirect (Mediated) Effects (bootstrap 95% CIs)**				
**Indirect path**	**b**	**95% CI (unstd.)**	**β**	**95% CI (std.)**
Identification → Hope → Resilience	0.0104	[0.0052, 0.0166]	0.131	[0.067, 0.196]
Identification → Optimism → Resilience	0.0056	[0.0027, 0.0093]	0.071	[0.034, 0.115]
Total indirect effect on Resilience	0.0160	[0.0089, 0.0236]	—	—

Note. R^2^ (Resilience) = 51.6%; R^2^ (Perceived Team Effectiveness) = 9.8%.

**Table 5:** Fit indices for both models (N = 269).

Fit Index	Overall Identification Model	Division of Labor Model
N	269	269
df	9	20
χ^2^	9.49	16.69
χ^2^ *p*-value	0.394	0.673
CFI	0.998	1.005
TLI	0.997	1.007
RMSEA	0.014	≈0
AIC	23.9	31.9
BIC	67.1	89.4

**Table 6:** Fit comparison: Division of Labor Model vs. Overall Identification Model (N = 269).

Fit Index	Overall Identification Model	Division of Labor Model
R^2^ (Resilience)	51.6%	51.6%
R^2^ (Perceived Team Effectiveness)	9.8%	12.1%

## Data Availability

The data that support the findings of this study are available from the corresponding author upon reasonable request.
